# Creating Advantages with Franchising in Healthcare: An Explorative Mixed Methods Study on the Role of the Relationship between the Franchisor and Units

**DOI:** 10.1371/journal.pone.0115829

**Published:** 2015-02-09

**Authors:** Karlijn Jojanneke Nijmeijer, Isabelle Natalina Fabbricotti, Robbert Huijsman

**Affiliations:** 1 Institute of Health Policy and Management, Erasmus University Rotterdam, P.O. Box 1738, 3000 DR Rotterdam, The Netherlands; 2 The Rotterdam Eye Hospital, P.O. Box 70030, 3000 LM Rotterdam, The Netherlands; University Hospitals of Geneva, SWITZERLAND

## Abstract

**Background:**

Franchising is a promising and increasingly used organizational form to improve strategic, organizational, professional and client-related results in healthcare. However, evidence is scarce regarding how franchises should be operated to actualize such results. This paper aimed to explore the association between the results achieved by healthcare franchises and the working relationships among actors in these franchises.

**Methods:**

A sequential mixed methods approach was used to obtain both in-depth and broader quantifiable insights into a little-investigated phenomenon. We first employed a qualitative multiple embedded case study. Data were collected through observations, document analyses, and 96 in-depth semi-structured interviews in three Dutch healthcare franchises. Within-case and cross-case comparative analyses were conducted. Subsequently, a cross-sectional survey was developed based on the qualitative study and disseminated among 19 healthcare franchises. 40 franchisors and 346 unit actors filled in the questionnaire.

**Findings:**

It seems important to have open, committed, cooperative franchise relationships in which professional franchisees and unit managers feel and trust that they have the opportunity to introduce ideas and articulate their needs to the franchisor. Such relationships help ensure satisfaction, survival, and quality of care, because they serve to foster synergy realization and local fit and prevent reinventing the wheel and professional resistance.

## Introduction

With the increasing prevalence of franchising as a form of inter-organizational cooperation in healthcare, it is important to understand what makes these franchise systems successful. However, although there is an abundant literature on the factors that influence the success of franchises in other industries, there is an absence of studies that focus specifically on healthcare [1, 2]. Studies in other industries suggest that how actors behave and feel with one another in franchise systems plays an important role in the success of such systems [e.g., 3, 4]. The aim of this study is to explore which relationship characteristics are related to success in healthcare franchises and why.

Franchising involves a contractual arrangement between two firms: the franchisor and the franchisee. In exchange for a payment, the franchisor offers a business format that consists of a brand name, support systems, and a specification of the services that must be delivered in local units. Entrepreneurial franchisees provide services in local units using the business format [5, 6]. In some franchises, certain units are owned by the franchisor and operated by employed unit managers who use the same business format as franchisees. Because the franchisor and the unit actors (franchisees or unit managers) have different roles and interests and must make the franchise system successful together, it is important to investigate the perspectives of both groups in the same study to investigate how franchises can be operated successfully [7].

Nowadays, in the USA there are at least 35 franchises that provide services related to home care, eye and hearing care, mental healthcare, care for the intellectually disabled, youth care, and (para)medical care (See, for example, http://www.entrepreneur.com/franchises/healthcare/indexhlth.html, http://www.bison.com/Healthcare_Franchises, http://www.franchisedirect.com/healthcareseniorcarefranchises/15, retrieved 20 December 2013). Approximately 21 such franchises are documented in the Netherlands, and there are at least 15 in each of the UK and Canada(see, for example, http://www.franchisesales.co.uk/search/care-services-franchise-health-care-franchises, http://www.franchisedirect.co.uk/carefranchises/175, http://canada.franchisesales.com/search/health-franchises, http://www.franchisedirectcanada.com/healthcare-senior-care-franchises-0505/, and http://www.canadafranchiseopportunities.ca/Senior-Care-Franchises/, retrieved 20 December 2013). 53 social healthcare franchises in various Asian and African countries were documented in 2011 [8].

The actors working in these franchises expect to realize a better competitive position, quality of care, efficiency, financial performance and professional work environment through shared positioning with a brand name and clearly defined services [9, 10], through supporting healthcare professionals in the units with operational support, proven practices and innovative developments in the business format and other units in the system [11, 12, 13], and through sharing knowledge and service innovations within the same formula [9, 10]. Difficulties may also arise as franchising requires uniformity to build a brand name and to achieve economies of scale, but this requirement may clash with professionals’ desire for autonomy [13].

Despite the increasing use of franchising in healthcare practices, there is hardly any scholarly research that can support practitioners in operating their franchise successfully (see recent systematic review [1]). This study aimed to gain insight into the characteristics of the relationship between the franchisor and unit actors (franchisees and unit managers) that are related to success in operating professional healthcare franchises. We also aimed to understand the underlying reasons for the importance of these characteristics to grasp how franchises can be managed effectively. We thus attempted to answer the following questions: 1) Which relationship characteristics are related to achieving positive strategic, organizational, professional, and client-related results in healthcare franchises? 2) Why are these characteristics related to results? (reasons) and 3) What are differences and similarities in the association between the results and the relationship characteristics from the perspective of franchisors, on the one hand, and unit actors on the other? By investigating two perspectives and multiple types of results within one study, we also contribute new insights to the broader franchise literature.

### Conceptual Model to Explore the Role of Relationship Characteristics in Healthcare Franchises

By applying relational exchange, relational marketing, organizational behavior, resource, and agency theories, studies in other industries have demonstrated the importance of various relationship characteristics associated with achieving positive results in franchise systems (see [2] for a systematic review). These studies have suggested that, in most cases, franchisors and franchisees are more likely to experience success, solid financial performance, competitive advantages, and satisfaction if they cooperate closely as partners with attuned tasks and attention to one another’s needs [14, 15, 16], if they trust one another [4, 17], if they frequently communicate and exchange information [3, 4, 18], if the level of conflict and opportunistic behavior in the system is low [18, 19], and if the franchisor and franchisees are committed to one another. The latter involves intentions and behaviors to increase long-term value for both [3, 17]. These studies in other industries hardly provided empirical insights regarding the underlying reasons for the importance of all these relationship characteristics. We used the insights from other industries as a conceptual model and for the genesis of our topic list to explore the role of relationship characteristics in professional healthcare franchises.

## Methods

### Study Design

**Fig 1:**
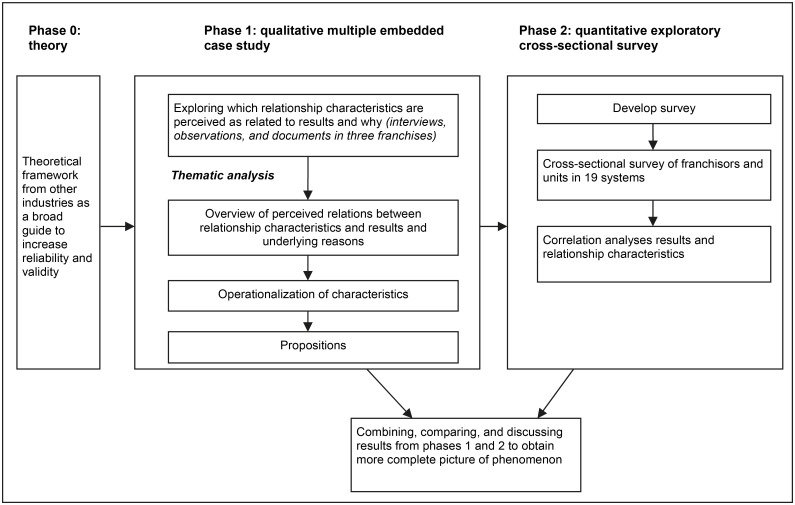
Overview of Explorative Sequential Mixed Methods Design.

We conducted a mixed methods study with an exploratory sequential design [20]. By combining qualitative and quantitative research, we quickly acquired a comprehensive in-depth and broad picture of an unexplored phenomenon in healthcare, and we were able to triangulate findings [21] (see Fig. 1 for an overview).

We began with a qualitative exploration in which the theoretical insights from other industries were used as a broad guide. The qualitative study had four purposes: 1) to identify the perceived relevant relationship characteristics in professional healthcare franchises to actualize positive results, 2) to understand the reasons underlying their relevance, 3) to develop propositions, and 4) to obtain operationalizations to develop survey measures. To achieve these purposes, a multiple embedded case study design was employed. We investigated three franchise systems in the Netherlands and sampled data from both the franchisor and the units. We used multiple cases to replicate findings and to identify diverging patterns. This increased the explanatory power and generalizability of our findings [22].

Subsequently, we developed a cross-sectional survey that was based on the qualitative study. This survey was disseminated among operational healthcare franchise systems in the Netherlands. The quantitative study had two purposes: 1) to explore the breadth of the findings and 2) to quantify the perceived relations between relationship characteristics and results.

### Phase 1: Qualitative Multiple Embedded Case Study

**Setting and Sample**. We conducted our study in the Netherlands, where approximately 21 healthcare franchises exist. We theoretically sampled our cases for the qualitative study from this group of franchises. We selected three franchises in different healthcare sectors and with different structures (existing organizations or individuals as franchisees) because scholars have assumed that such differences may play a role in how actors behave and which results are achieved [13]. The three cases provide mental healthcare (system 1), hospital eye-care (system 2), and care for the intellectually disabled (system 3). A description of the cases is provided in Table 1. As shown in this table, the franchisors of all three cases started franchising to improve the quality and efficiency of care for idealistic and/or competitively instrumental reasons. The franchisors are existing healthcare organizations (system 1 and 2) or an individual entrepreneur (system 3) that started-up a new or alternative organizational model. All franchisor bodies work with one or more directors and a support staff. The franchisor bodies developed a business format that, among others, includes knowledge, branding materials and work methods laid down in an operations manual. In return for a fixed or variable payment, the franchisors manage the system, provide the business format to units and support them to a certain degree in applying the business format appropriately. In principle the franchisees and unit managers operate their local unit(s) by making use of the business format, supplemented by their own activities and ideas. Sometimes such local activities and ideas are adopted by the franchisor and included in the business format. Professionals, who regularly also assume the franchisee or unit manager role, are psychologists, psychiatrists (system 1), ophthalmologists (system 2) or professional care providers for intellectually disabled persons (system 3) (see table 1).

**Table 1:** Description of Cases Used in the Qualitative Study.

	System 1	System 2	System 3
***Background information***			
**Service**	Mental healthcare (specialized ambulatory care to adults)	Hospital care (eye-care)	Care for the intellectually disabled
**Year of establishment**	2004	Franchise since 2007, system started in 2003	2003
**Motive for franchising**	Gain stronger position in more competitive market through high-quality, efficient care	Gain stronger position through provision of high-quality efficient care in increasingly competitive market	Founded by a father who was highly dissatisfied with the quality of regular care for his intellectually disabled son
**Number of units**	26 owned by 4 franchisees.	14; 11 franchised and 3 owned by the franchisor.	107; 99 franchised and 8 owned by the franchisor.
**Payment method of care provided in units**	(Obligatory) health insurance reimbursement, complemented with personal contribution of clients. System is obliged to work not-for-profit under Dutch law.	(Obligatory) health insurance reimbursement, complement with personal contribution of clients. System is obliged to work not-for-profit under Dutch law.	Personal budget of clients provided by governmental regional care offices following the Exceptional Medical Expenses Act. System is allowed to work for-profit under Dutch law.
***Description of franchisor, franchisees and unit managers***			
**Franchisees and unit managers**	Mental healthcare organizations are franchisee; a portion of their care delivery is franchised. Units are daily operated by unit managers, of which some also have a professional role. Professionals are psychologists or psychiatrists who do not own the franchise (are employees).	Eye care departments of general hospitals are franchised. Three departments are owned by the franchisor and operated by employed unit managers. Professional ophthalmologists work in a partnership or as employees.	Two professional caregivers for intellectually disabled persons own and operate a small-scale full-time living facility as a franchisee, or fulfill the same task as an employed unit manager.
**Franchisor representatives**	Four mental healthcare organizations are shareholder-franchisor (as well as franchisee). The franchisor body is daily operated by directors of these organizations and their support staff.	Director and support staff of a private liability company of a specialized ophthalmology hospital.	Owner (founding father), director and support staff.
***Business format and contractual payments in the franchise***			
**Business format**	Franchisor developed business format which included branding, shared access system, operations manual, marketing, intranet, outcome measurement and knowledge-sharing meetings.	Includes branding, operations manual, intranet, marketing, benchmarking, knowledge-sharing facilities, training, shared purchasing, ongoing advisory support	Includes branding operations manual, initial training, facilitation of care building and a loan, administration system, quality measurement, advisory support.
**Contractual payments**	All franchisees are shareholder of the franchise. All costs are proportionally divided and paid.	Fixed initial fee for quick scan/research before joining franchise. Ongoing annual fee comprising fixed base fee + variable fee per FTE ophthalmologist.	Fixed initial fee and fixed annual ongoing fee.

**Data Collection**. Data were collected between 2009 and 2012 from observations, strategic and operational archival documents, and 96 interviews with franchisors, franchisees, and unit managers. In the interviews, we could ask respondents about their experiences and perceptions to gather rich data about the actual behaviors of and interactions among actors, the perceived effect on results, and the mechanisms underlying these perceived effects. A predetermined topic list based on theory from other sectors was used as a broad guide to increase reliability and validity. Respondents had ample opportunity to introduce topics and reflections. The interviews lasted on average 1.5 hours and were recorded and transcribed verbatim. Documents were used to prepare the interviews and to complement and triangulate the interview findings. Observations of meetings and units were used to stimulate new lines of inquiry, triangulate the data, and obtain additional insights by observing the actual behavior of and interaction among actors (e.g., observing how a franchisor and its franchisees exchange information).

**Data Analysis**. The data were thematically analyzed in Dutch. Themes were derived both deductively and inductively. First, transcripts and documents were read several times while primarily deductively applying codes to the data using theory derived from other industries. Second, we used within-case comparison techniques to further conduct the analysis for each code. We inductively applied new codes to the data to refine the analysis. The resulting analysis was recorded in a case report per franchise system that was checked with case representatives to verify and complement the analysis. Third, we searched for consistent and distinct patterns among cases. This analysis resulted in an overview of the relationship characteristics and sub-characteristics that were perceived to be important to achieving positive results and the underlying reasons for their importance. Differences in perceptions between franchisors, franchisees and unit managers and across systems were noted.

### Phase 2: Quantitative Cross-Sectional Survey

**Sample**. All operational Dutch franchise systems in hospital care, mental healthcare, home and elderly care, paramedical care, care for the intellectually disabled, and youth care were asked to participate in the cross-sectional survey (n = 21). Two systems decided not to participate due to time constraints and internal conflicts. Ultimately, 19 franchises were included that vary in age and size (see table 2). Within each system, franchisor representatives (n = 44) and unit actors (franchisees and unit managers) (n = 518) were asked to fill in a questionnaire sent by mail in June 2013. The franchisors urged their franchisees and unit managers several times to participate in the study. Three weeks after sending the survey, we sent a reminder to all non-respondents. Three weeks thereafter all non-respondents received the questionnaire again. These non-respondents were given three additional weeks to respond. This procedure resulted in a sample of 40 franchisors (91% response) and 347 unit actors (67% response). Similar to the characteristics of the three qualitative cases, these franchisor representatives are owner and/or director of the franchise organization or a major member of the support staff that manages and supports the unit actors. Similar to the qualitative cases, unit actors are individual entrepreneurs with a care provider role, or managers and professionals of franchised organizations. Depending on the sector, healthcare professionals are physicians (mental healthcare, hospital care), psychologists (mental healthcare), nurses and professional care practitioners (home and elderly care, youth care and care for intellectually disabled), or paramedics (e.g., physiotherapists). Because our qualitative study did not reveal clear patterns regarding differences in views on the importance of various relationship characteristics on the one hand, and differences in contracts, business format characteristics or sector differences on the other hand, we did not attend to any of such differences in our quantitative study.

**Table 2:** Characteristics of Franchises (n = 19) Participating in Quantitative Study.

Sector	Mental healthcare (n = 5)
	Hospital care (n = 1)
	Home care and elderly care (n = 8)
	Paramedical care (n = 2)
	Care for intellectually disabled and youth care (3)
Size	1–10 units (n = 7)
	10–30 units (n = 6)
	> 30 units (n = 7)
Age of system	1–5 year (n = 9)
	6–10 year (n = 7)
	> 10 year (n = 3)

**Data Collection**. A preliminary formulation of the survey items emerged from the qualitative study. Items were reformulated into existing (validated) items from the franchise literature if they measured the qualitative operationalization appropriately. Subjective (i.e., perceptual) metrics—instead of objective metrics—were used to measure the results for two reasons. First, objective data (e.g., about quality and efficiency of care) were impossible to obtain in all systems. Second, different sectors are more easily compared using perceptual measures [23]. The use of subjective measures has been shown to be an effective and valid substitute for measuring ‘objective’ results [24, 25]. All items but two were measured on the commonly used five-point Likert scale (1 = totally disagree, 5 = totally agree) [26], plus the categories ‘don’t know’ and ‘not applicable’. The surveys were piloted in a meeting with representatives of the three case-study systems to check for clarity, length, ambiguity, and usefulness of the five-point Likert scale. This pilot led to several changes. The items were formulated in Dutch and translated into English for drafting this paper. All items are included in S1 Appendix.

**Data Analysis**. Data about relationship characteristics were analyzed at the item level to allow full integration with the in-depth qualitative study and to acquire a rich explorative analysis (scales would imply a reduction of the richness of data). Data about results were reduced to scales because the qualitative study indicated that a global measure for every result type (e.g., quality of care) was sufficient for our study that was focused on relationship characteristics. A scale was constructed when the Cronbach’s Alpha was >.60 or when the inter-item correlation was >.30 for a scale with two items. These measures are reported in S1 Appendix.

Data were analyzed using SPSS 19.0. First, for both actor groups, frequencies were calculated for all variables. Variables with more than 10% missing values (‘don’t know’ or system missing) were excluded. The result ‘intend to remain’ was excluded in the unit group (19.5% missing). In the unit group, one respondent was excluded because all answers were missing (total n = 346). Second, means and standard deviations were calculated for all items and scales in both actor groups. Third, because our measures produced ordinal data, correlations among result types and relationship items were calculated with Kendall’s tau_b [27]. Cases with missing values were excluded pairwise. Because of the relatively small size of the franchisor group (n = 40), a correlation was considered significant at the level of p<0.1 (two-tailed). Correlations were considered to be weak between ±.10 and ±.30, moderate between ±.30 and ±.50, and strong above ±.50 [28]. To appropriately answer our research question the analysis primarily attended to correlations with a strength of > ±.30.

### Ethics Statement

This study took a managerial perspective and thus only involved data sampling from managers and professionals. We did not have access to any medical or other identifying information to clients of the participating organizations. Because no medical data was used and patients were not involved in any way, no ethical approval of our Institutional Review Board nor written informed consent was required (see formal criteria Erasmus Medical Center, Medical Ethical Committee, http://www.erasmusmc.nl/commissies-cs/metc-cs/573270/reikwijdte [in Dutch]. Formal approval is only required if 1) it concerns medical research involving patients, and 2) if persons are subjected to particular treatments or must undertake particular behaviours). We observed the European code of Conduct for Research Integrity. All the franchisors, franchisees and unit managers who were selected for interviews received a letter with information about the purpose, content, and reporting about the study prior to the interview. They were asked for consent to participate based on this information prior to interview planning. When they refused participation the interview was not conducted. At the start of each interview verbal consent was asked for recording the interview. Recording was omitted when consent was not obtained. The interview transcripts where stored anonymously with function title and number of the participating organization only, and all names in the interviews were made anonymous in a similar way. To ensure anonymity in the analysis and reporting of the data only broad function titles and organization numbers were used (e.g., franchisee system 1). Observations of meetings were only conducted when all participants agreed. We had the work addresses and names of potential survey respondents to send the questionnaires. They received a letter with study information in the same envelop. Based on the letter they could decide not to fill in and send back the survey. To ensure anonymity each questionnaire only had a number to identify the responding franchise system; respondents were not asked to fill in their names or other identifying information. Survey data were stored with this anonymous number only.

## Results

**Fig 2:**
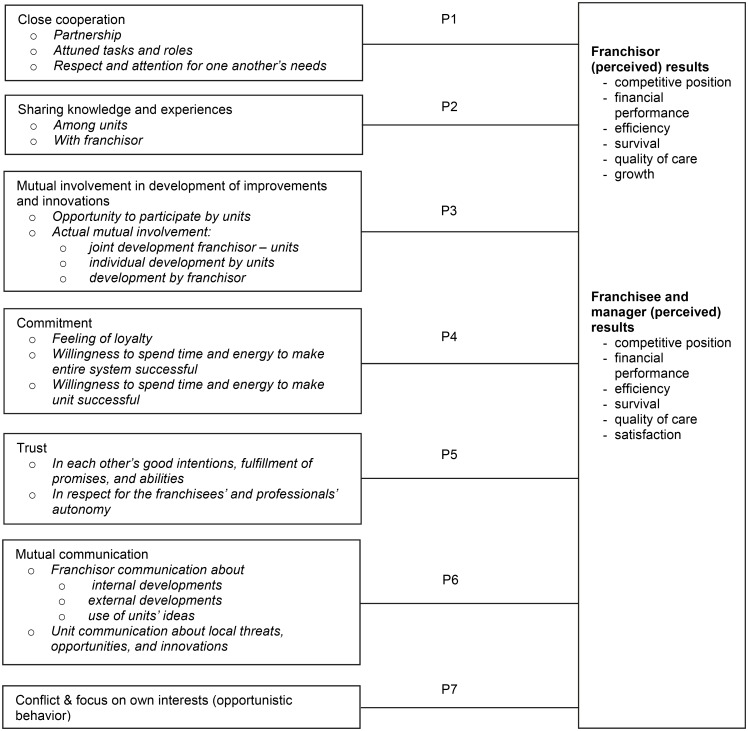
Theoretical Framework Based on Qualitative Case Studies to Explore in Quantitative Survey Research.

The analysis of our qualitative study revealed that seven aspects are perceived to be important to actualize positive results in healthcare franchises: close cooperation, mutual involvement in the development of improvements and innovations, sharing knowledge and experiences, mutual commitment, trust, mutual communication, and limited amounts of conflict and opportunistic behavior (see Fig. 2). This section describes why these aspects are perceived as success factors and explores whether these perceived relationships were confirmed in the cross-sectional survey research.

### Close Cooperation

Consistent with our conceptual model, all respondents highlighted the stimulating effects of close cooperation among actors in the franchise system. This indicates three aspects in franchisor-unit actor relationship. First, giving attention and respect to one another’s needs and wishes was perceived to promote results because it reduces the chance of conflict and professionals’ resistance to change, in addition to preventing the development and implementation of elements in the business format that are not valuable to local units: “*The franchisor sometimes develops policies…and we say*, *what are you doing*? *This is not our need on the shop floor*. *Please guarantee that what you develop fits with our needs” (franchisee)*.

Second, successful franchising in healthcare is perceived to require partnership rather than a hierarchical controlling attitude of the franchisor. When the latter is employed, professionals will not be sensitive to improvements suggested by the franchisor and will resist change: “*You have to manage much more on the relationship with units (than in retail)*, *talk with the franchisee*, *and then (checklists and things alike) are considered as well*, *but you first have to make sure that you have an egalitarian relationship*. *Otherwise*, *nothing will happen” (franchisor)*. Moreover, when the franchisor and units act as partners by combining their strengths and inputs (i.e., the franchisor’s business format and the unit’s local experiences), they realize the most synergy. As one franchisee stated, “*It is a combination of the format and experience of (the franchisor) and our own input; it is not so black and white*, *that you can say this is my share*, *and this is your share*. *I could never have done it alone; it really requires a partnership to make it successful*.”

Third, well-attuned tasks and roles were perceived to be important to prevent reinventing the wheel and to realize optimal synergy in the franchise system. Such attuning of tasks and roles implies that franchisors really employ the franchisor role in offering a business format and managing the system and that units adequately apply the content of the business format in their unit and supplement it with their local experiences: “*As a franchisor we have to manage and provide the formula*. *(…) and the franchisees are putting it into practice; if they were not there and were not fueled by our formula and ambition*, *then it would not be successful” (franchisor)* Attuning of tasks and roles is also important in the entire system between units to create synergy and prevent reinventing the wheel: “*I have the impression that we still invent the same wheel at various locations; we are not well attuned in this system while that is important to realize advantages” (franchisee)*. These qualitative findings lead to the following proposition (see S1 appendix for developed measures):

P1 Close cooperation—i.e., attention to needs and wishes, partnership, attuned tasks and roles—is related to results.

The quantitative study showed consistent patterns of positive correlation coefficients (except for one coefficient) between results, on the one hand, and attention to needs, partnership, and attuning tasks, on the other, in both the franchisor and the unit groups (see table 3, row A). However, moderately strong, significant correlations were found with only certain result types. Unit actors are more satisfied and likely to promote working in the franchise if they perceive more attention regarding their needs, partnership, and attuning (all τ ≥ .33, p <.001). Those unit actors that experience more attention regarding their needs also rate their survival chances higher because of participating in the franchise (τ = .36, p <.001). The latter was also found in the franchisor group (τ = .36, p <.05), as was a relation between survival and attuning tasks (τ = .32, p <.05). Attuning tasks was also moderately related to the franchisor’s perceived quality of care (τ = .30, p <.001).

**Table 3:** Qualitative and Quantitative Relations Between Relationship Characteristics and Results.

Qualitative study			Quantitative study								
Relationship characteristics related to results (perceived)		Actor	Financial results good	Efficiency of care and innovation	Quality of care		Competitive position	Survival chance of participant	Satisfaction		Growth system
					Good quality care (scale)	Promote care (NPS)			Satisfied with work in franchise (scale)	Promote work in franchise (NPS)	
A: Close cooperation	Attention needs	FO	.05	.08	.26#	.04	.17	.36*	-	.19	.11
		Unit	.19***	.11*	.20***	.26***	.23***	.36***	.42***	.46***	-
	Partnership	FO	.25#	.15	.18	.01	.17	.22	-	.14	.12
		Unit	.14**	.07	.11*	.17***	.15**	.26***	.33***	.38***	-
	Attuning tasks	FO	-.02	.20	.30*	.20	.06	.32*	-	.20	.26#
		Unit	.17***	.11*	.14**	.25***	.15**	.27***	.38***	.39***	-
B: Sharing knowledge & experiences	Between units	FO	.09	.12	.32*	.21	.24#	.39**	-	.34*	.21
		Unit	.15**	.21***	.17***	.20***	.17***	.12*	.17***	.17***	-
	With franchisor	FO	.19	.21	.29*	.10	.11	.21	-	.29*	.17
		Unit	.05	.05	.01	.07	.01	.05	.04	.09*	-
C: Mutual involvement in developing innovations & improvements	Opportunity participate	FO	-.04	-.09	-.04	.14	.02	.30*	-	.32*	.19
		Unit	.12*	.11*	.08	.10*	.12**	.18**	.31***	.28***	-
	Unit development	FO	.12	-.17	-.10	-.33*	.07	-.06	-	-.13	-.22
		Unit	.16**	.22***	.14**	.10*	.18***	.12*	.05	.11*	-
	Joint development (think along)	FO	-.08	-.04	-.04	-.04	.04	.45**	-	.25#	.27*
		Unit	.05	.05	.02	.09	.08	.08	.09*	.14**	-
	Franchisor develop	FO	-.12	-.05	-.02	-.02	-.12	.24#	-	.13	-.02
		Unit	.06	.03	.08	.04	.02	.11*	.24***	.15**	-
D: Mutual commitment	Loyalty	FO	-.07	.22	.27#	.45**	.19	.25	-	.32*	.22
		Unit	.19***	.22***	.26***	.44***	.29***	.36***	.40***	.43***	-
	Willingness to make system successful	FO	-.07	.18	.31*	.42**	.21	.27#	-	.43**	.15
		Unit	.12*	.17***	.26***	.31***	.26***	.30***	.27***	.31***	-
	Willingness to make unit successful	FO	.19	.34*	.26#	.31*	.22	.38*	-	.43**	.35*
		Unit	.19***	.25***	.33***	.24***	.27***	.33***	.24***	.23***	-
E: Trust	Franchisor-unit trust	FO	.06	.19	.49**	.18	.18	.33*	-	.24#	.24#
		Unit	.22***	.09*	.18***	.33***	.21***	.38***	.48***	.50***	-
	Trust respect autonomy	FO	.18	.22	.51**	.45**	.39**	.42**	-	.26#	.25#
		Unit	.20***	.13**	.22***	.26***	.20***	.34***	.38***	.42***	-
F: Mutual communication	By unit	FO	.08	.29*	.43**	.13	-.02	.15	-	.10	.10
		Unit	.15**	.12*	.15**	.22***	.14**	.23***	.20***	.23***	-
	About external development	FO	.06	-.01	.29*	.07	.11	.26#	-	.16	.30*
		Unit	.12*	.12**	.18***	.16***	.16**	.18**	.31***	.27***	-
	About internal developments	FO	.09	-.04	-.06	.07	.14	.36*	-	.23#	.26#
		Unit	.08	.07	.10*	.09*	.08	.17**	.29***	.28***	-
	About what is done with ideas	FO	.04	.12	.05	.24#	.13	.24#	-	.30*	.24#
		Unit	.07	-.11*	-.03	.07	.05	.08	.18***	.26***	-
G: Conflict, opportunistic behavior	Conflict	FO	-.30*	.02	.11	.45**	.06	-.01	-	.22	.21
		Unit	-.16**	-.05	-.04	-.10*	-.13**	-.18**	-.29***	-.30***	-
	Focus own interests	FO	-.20	-.09	-.26#	-.04	-.03	-.17	-	.04	-.07
		Unit	-.18***	-.12**	-.13**	-.29***	-.20***	-.30***	-.43***	-.41***	-

# P < .10;

* P <.05;

** P<.01;

*** P<.001.

FO (n = 40) = franchisor; Unit (n = 346) = franchisees and managers operating units under the business format.

### Sharing Knowledge and Experiences

The franchisors and certain unit actors (franchisees and unit managers) in our healthcare cases also perceived that sharing knowledge and experiences promotes the actualization of positive results; an insight not indicated by our conceptual model. Both sharing between units and sharing with the franchisor are considered to be important in healthcare franchises for three reasons. First, improvements and innovations are believed to be realized more quickly in the units than if units were operating alone because not every unit must reinvent the wheel: “*Many hospitals (not operating in a franchise) are reinventing the wheel; I find that strange because many hospitals have the same kind of problems*. *You can learn a lot from the solutions of others*. *So it is a big advantage to learn from others in the system” (franchisee)*. If improvements are also shared with the franchisor, they spread more easily to other units in the system: “*If they have developed a nice product or improvement we make it generic and put it into the operations manual*, *so that everyone in the franchise can make use of it” (franchisor)*. Second, multiple unit actors felt relieved by quickly obtaining knowledge from other units and consider this to be one of the major advantages of franchising: “*For example*, *if you need a diabetes protocol to improve your organization of the care process*, *you email or make a phone call to another (unit) or the franchisor*, *you turn around three times*, *and you have it*!” Third, multiple unit actors find it pleasant to make use of the experience and expertise of peers from other units in the franchise system and to have sparring partners: “*Sometimes I call a colleague-franchisee*, *and say*, *I have these difficulties with my personnel*, *how would you handle that if you were me*? *Do you think I can do this*? *It is very pleasant that you can share such things with one another*, *you can learn*, *I think that is very interesting” (franchisee)*.

However, not all unit actors perceive that knowledge sharing within the franchise promotes their results. Some do not feel the desire to make use of the knowledge of other units in the system in addition to the knowledge included in the business format, and do not make time to share: “*We are just nicely operating our unit here*, *(knowledge sharing) is not high on our priority list*. *If we really have questions we contact the franchisor” (franchisee)*. Some also believe that the knowledge of others in the franchise does not add value to their existing knowledge structures with professionals or organizations outside the franchise system: “*We already had conferences with our professional group in the region where we shared knowledge*, *did research*, *etc*. *So sharing with others in the franchise does not add anything*, *it is just the same*, *sometimes even on a lower quality level than we do in the region” (franchisee)*.

These qualitative findings lead to the following proposition:

P2 Sharing knowledge and experiences—i.e., sharing between units, with the franchisor—is positively related to franchisor results and positively or unrelated to unit results.

Consistent with the franchisors’ qualitatively perceived importance of both types of knowledge sharing, a consistent pattern of positive correlation coefficients was found (see table 3, row B). However, moderately strong correlations (± >.30) were only found with franchisors’ perceived quality of care (τ = .29, .30, p <.05) and franchisors’ perceived attractiveness of working in the system (i.e., the propensity to promote work) (τ = .34, .30, p <.05). Moreover, more knowledge sharing between units was related to enhanced survival (τ = .39, p <.01). Only weak positive correlations were found for units (τ ≤ .20), which may be consistent with the unit actors’ difference of opinions in the qualitative study.

### Mutual Involvement in the Development of Improvements and Innovations

In addition to the relationship characteristics indicated by theory, our qualitative study in healthcare showed the importance of a relationship in which the franchisor and unit actors are mutually involved in the development of improvement and innovations in the franchise system. Specifically, such mutual involvement indicates that both the franchisor and the units are involved in developing new and adapted strategies, policies, services, and work methods in the franchise system. Involvement of units—either by jointly developing innovations and improvements with the franchisor or by developing them in the individual unit—is perceived to promote results for three reasons. First, this involvement increases the chance that adaptations and innovations to the business format are feasible and really promote the results of units: “*By adding your own input*, *you maintain your own culture*, *your philosophy*, *you do what fits within your own (unit)” (franchisee)*. Second, healthcare professionals feel more ownership and feel respected in their professional ideas. This also results in a reduction of their resistance to change: “*Professionals in units do not like to be dictated about how to work*, *they want to have influence by themselves*. *The franchise organization as it is now allows professionals to share in the development of care programs; together*, *they can think about how to improve protocols” (manager)*. Third, the innovation and adaptation process in the franchise system proceeds faster and is more powerful when both the franchisor and the units are involved: “*(The process of developing together) can be more intense*. *I have the feeling…the way it was originally invented was super*, *but it has not been entirely implemented in this manner*. *I think it is therefore less powerful than it could be” (franchisee)*.

However, not all franchisees and unit managers want to invest time or feel the desire to be involved in developing improvements and innovations. They find it important that they have the opportunity to participate but do not exploit that opportunity in practice: “*Not everyone wants to think about new work methods; if you say*, *give me some help thinking*, *some say ‘I do not want that*!*’ So it is not so black and white” (franchisee)*.

Respondents felt that the franchisor should also develop many improvements and innovations to the business format to promote positive results, for several reasons. First, more development by the franchisor allows units to devote more time to actually providing care to clients. Second, franchisees can be dissatisfied with the value of the franchise and the fees paid if they perceive that the franchisor’s involvement is too low: “*We had the feeling in that project that we were inventing it*, *we wrote documents that the franchisor would literally use in other (units)*, *while we thought*, *that is your task*! *I am not going to produce policy documents (…) (those) should be developed by the franchisor” (franchisee)*. Third, franchisors believe that too-heavy of an involvement of units reduces the franchisor’s control in maintaining uniformity in the brand. These qualitative findings lead to the following proposition:

P3 Mutual involvement in the development of improvements and innovations—i.e., the units have the opportunity to participate and there is actual development by both units and the franchisor—is positively related to results.

As was expected from the qualitative study, units were significantly more satisfied and likely to promote working in the franchise if they had more opportunities to participate in development (τ = .31, .28, p <.001), whereas their actual level of involvement mattered less for their satisfaction (see table 3, row C). Although a pattern of positive correlation coefficients was found in units between all result types and the actual level of unit development, joint development and franchisor development—which was expected—moderate or strong correlations were surprisingly absent. From the franchisor perspective, by contrast, joint development was moderately positively related to the survival chance of participants (τ = .45, p <.01). Moreover, as opposed to the positive pattern in units, an almost consistent pattern of negative correlation coefficients was found for franchisors with respect to development by units, in which only a moderate correlation with propensity to promote care was significant (τ = -.33, p <.05).

### Mutual Commitment

Respondents also highlighted the stimulating effects of committed franchisor representatives and unit actors. Three elements were perceived to be stimulating: a feeling of loyalty, willingness to devote time and energy to make the system successful, and willingness to devote time and energy to make the unit(s) successful. The mutual commitment of all actors to both the system and units is a further detailing of the mutual commitment concept found in other industries. We found various underlying reasons for these perceived stimulating effects. First, an expressed feeling of loyalty of actors was perceived to help protect the system from reputational harm, build a competitive advantage, and achieve system growth: “*They always speak positively about us when they talk with other (organizations)*. *That also really helped us to acquire new franchisees” (franchisor)*.

Second, respondents felt that the franchisor’s willingness to devote time and energy to making the system successful promotes better results because it indicates that the franchisor is making an effort to keep the business format valuable and competitive. Franchisees’ and managers’ willingness to devote time and energy to the system’s success was perceived to promote results because it augments the chances that units will develop and share improvements and experiences with others in the system. *“Some franchisees are very committed to the franchise*. *They are willing to promote it to other franchisees and help and educate them in how to adequately operate the franchise formula in the unit*. *(…) We like them more” (franchisor)*.

Third, respondents argued that the willingness of franchisees and unit managers to devote time and energy toward making their unit successful—which indicates that they are motivated, that they proactively attempt to ensure fit with the local context, and that they are willing to change toward better working methods developed by the franchisor or other unit actors—plays an important role in the eventual results. Passively applying the business format is not sufficient; effort and input of actors in appropriately applying and complementing the business format insights to the local situation is pivotal: “*If you put substantial energy into it*, *you can really achieve much success in this unit*, *but you have to do it yourself” (franchisee)*. Various respondents also found it important that the franchisor was willing to devote time and energy toward making individual units successful because doing so speeds up the improvement and implementation processes and unburdens care professionals in units from doing non-care related tasks. However, franchisors and some of the units felt that the franchisor’s commitment to units should not be too great relative to the time and energy unit actors devote in their unit because this undermines the regular task division between the franchisor and unit actors in franchising, rendering the system inefficient. *“Sometimes there are new franchisees that directly refer difficulties back to us*, *then we think*, *can we get rid of these franchisees*? *Because…that is employee behavior*, *then they should have stayed employed*. *It has become your own enterprise*, *you should really go for it yourself*!*” (franchisor)*. These qualitative findings lead to the following proposition:

P4 Commitment—i.e., a feeling of loyalty and the willingness to devote time and energy for the success of the entire system and the unit(s)—is positively related to results.

Except for two negligibly small negative coefficients, consistent patterns of positive correlation coefficients were found in both the franchisor and unit groups with all commitment elements (see table 3, row D). However, correlations were significant and moderately strong only for certain result types. Franchisors and unit actors with stronger feelings of loyalty and willingness to make the system successful are more likely to promote care and work in the system (all τ ≥.31). Units also rate their survival chances to be higher (τ = .30, .36, p<0.001). Units feel more satisfied when they sense loyalty (τ = .40, p<.001), and the franchisor’s willingness to make the system successful was moderately related to the quality of care (τ = .31, p<.05). The willingness of the franchisor and the units to make individual unit(s) successful was moderately related to the survival chance of both parties and to a quality of care measure (all τ ≥.31). Moreover, franchisors are more likely to promote working in the system (τ = .43, p<.01) and to rate their system growth (τ = .35, p<.05) and (somewhat surprisingly) efficiency of care (τ = .34, p<.05) more highly.

### Trust

Consistent with our conceptual model, a high level of trust was consistently perceived as important to actualize positive results. Franchisors and units must trust in one another’s abilities, good intentions and trustworthiness to keep promises. Moreover, our study in the professional healthcare sector indicated that unit actors must trust that the franchisor respects their professional autonomy. Our study reveals that these types of trust are perceived to be important to obtain positive results for four reasons. First, respondents argued that unit actors resist franchisor actions and proposals for change when they suspect that the franchisor may not respect their professional autonomy. Second, when either the franchisor or the units do not believe that the other party keeps its promises, they are less inclined to make efforts to benefit the shared or the other party’s interests. Third, without trust, actors do not want to share relevant information: “*Some franchisees think ‘you are going to use (the information) against us’*. *(…) And (say) ‘what happens when I give this to you*? *I lose control’!” (franchisor)*. Such reluctance to share makes it difficult to develop improvements jointly and prevents franchisors from developing suitable support to units. Fourth, tasks and roles can be divided more efficiently when the franchisor and franchisees trust one another because both parties are giving up some control. *“In principle*, *you can provide substantial freedom in the franchise arrangement*. *You have to trust the other*, *you have to dare to let go*. *Then*, *you can work with very low overhead costs” (franchisor)*. These findings lead to the following proposition:

P5 Trust—i.e., franchisor-unit trust and trust in professional autonomy—is positively related to results.

As expected from the qualitative study, consistent patterns of positive correlation coefficients were found with both types of trust in both actor groups (see table 3, row E). However, moderate to strong correlations were found only with quality of care measures, survival, competitive position, and satisfaction measures and—surprisingly—not with efficiency and financial performance. The moderate correlations with satisfaction measures were found only from the unit perspective (τ ≥ .38, p<.001). Competitive position was moderately correlated only in the franchisor group, and only with trust regarding professional autonomy (τ = .39, p<.01).

### Mutual Communication

Consistent with findings from other industries, respondents in our study argued mutual communication to be important. We found that mutual communication indicates that units communicate with the franchisor about the threats, opportunities and innovations in their localities and that the franchisor communicates about internal and external developments and about using ideas proposed by the units. Our respondents perceived communication by units to promote positive results for two reasons. First, it prevents the franchisor and other units from investing time in developing similar improvements. Second, this information helps franchisors adapt and strengthen the business format: “*I believe it is important to share with the franchisor what I see happening here in [the unit] and the difficulties I am confronted with; that keeps our business format up-to-date and focused on the right things*. *Then*, *I write an A4 to the franchisor*, *so you can contribute to keeping the business format strong” (franchisee)*.

Various types of communication by the franchisor to the units are also felt to be important, primarily by franchisees and managers. First, franchisor communication about external developments (e.g., changing governmental financing) is thought to help units anticipate changes. Second, communication by the franchisor about developments at headquarters and in units across the system (e.g., innovations, marketing efforts) helps maintain the satisfaction of the franchisees because it informs franchisees about the value of the franchise fees: “*Many things they do are not directly visible*, *you will not notice them right away*. *(…) and then when you see what they have really done*, *you think*, *oh we could not have done that without them” (franchisee)*. Third, communication about changes in the business format or contract is thought to prevent conflicts because it creates understanding of changes that are unpleasant or are experienced as an infringement on autonomy. *“The issue of autonomy*. *It really has something to do with working with professionals*. *However*, *it also has to do with communication*. *You have to make clear that intervention does not reduce autonomy because they feel it like that” (manager)*. Fourth, unit actors argue that the franchisor should communicate how it has used the ideas they have proposed to maintain their future enthusiasm in participating in development: “*We missed that*. *It is not very motivating to put effort again in a project if we do not hear back or see anything about it” (franchisee)*.

However, franchisors did not want to communicate as much as many unit actors desired. To maintain system efficiency and manageability, franchisors find it primarily important to communicate about things that directly impact the units’ operations and about sensitive topics to reach the necessary acceptance levels: “*We are an efficient*, *agile organization; the franchisees should not have to know about and discuss everything*. *If you inform them about everything and make it a democratic process*, *you will not achieve any change*.” These findings lead to the following proposition:

P6 Mutual communication—i.e., units communicating about local developments, franchisor communicating about use of ideas and internal and external developments—is positively related to results.

As expected from the qualitative study, consistent patterns of positive correlation coefficients were found for communication by units about local developments (except for one negligibly small negative coefficient of -.02) for both actor groups (see table 3, row F). However, moderately strong correlations were only found in the franchisor group with perceived quality of care (τ = .43, p<.01) and efficiency of care and innovation (τ = .29, p<.05). In the unit group, surprisingly, all correlations were significant but weak.

Consistent with the qualitative findings, communication by the franchisor about internal and external developments was primarily positively related to satisfaction with the franchise and propensity to promote work in the unit group (τ = .27 to .31, p<.001), whereas communication about the use of proposed ideas was weakly to moderately related to the latter satisfaction measure (τ = .26, p<.001) (see table 3, row F). From the franchisor perspective, communication about external developments was moderately related to growth (τ = .30, p<.05) and perceived quality of care (τ = .29, p<.05), communication about internal developments was related to survival (τ = .36, p<.05), and communication about the use of ideas was moderately related only to the propensity to promote work (τ = .30, p<.05).

### Conflict and Opportunistic Behavior

Consistent with our conceptual model, our healthcare cases reveal that conflicts between the franchisor and units can hinder the achievement of positive results, as can a strong focus on self-interests. Four reasons seem to underlie this hindrance in healthcare. First, a strong focus of actors on their self-interests and high conflict levels take time and energy away from undertaking cooperative activities in the franchise system that are important to strengthening results. As a franchisee stated, *“You can fight because you do not want particular things*, *but if that continues you kill off the entire system and that is not the purpose*.” Second, respondents argued that a dominant focus on self-interests and high conflict levels lowers trust and commitment levels in the system with subsequent negative effects. Third, such a situation is perceived to have a negative effect on the atmosphere and work environment. Fourth, conflict and a tendency to focus on self-interests indicate that the shared or individual interests of actors can be harmed. Such a situation is perceived to lower satisfaction and performance. *“People continuously monitor what their self-interests are*, *and if they have any chance*, *they just do what is good for their self-interests*. *That is unfortunate for [the franchise]” (manager)*. These qualitative findings lead to the following proposition:

P7 Conflict and a heavy focus on self-interests are negatively related to results.

Consistent with the qualitative findings, in the unit group, consistent patterns of negative correlation coefficients were found between results, on the one hand, and the level of conflict and focus on self-interests, on the other (see table 3, row G). However, moderately strong negative correlations were found only with satisfaction and propensity to promote work (τ = –.29 to -.41, p<.001), and for a focus on self-interests also with survival (τ = -.30, p<.001) and propensity to promote care (τ = -.29, p<.001). In the franchisor group, by contrast, both negative and (surprisingly) positive coefficients were found. More conflict was even moderately positively (τ = .45, p<.01) related to the propensity to promote care. A moderately negative correlation was only found between conflict and financial performance (τ = -.30, p<.05).

## Discussion

### The Role of the Relationship Between the Franchisor and the Units

The qualitative study showed fairly unambiguous perceptions of franchisors and units regarding which relationship characteristics are related to results and how. Both actor groups perceived a close cooperation with attention for one another’s needs, partnership, and attuning one another’s tasks to be important for achieving positive results with franchising, in addition to mutual commitment to the entire system and individual units, a trustful relationship, the opportunity for units to participate in developing improvements and innovations, mutual communication, and low levels of conflict and opportunistic behavior. Franchisors also found that knowledge sharing and a certain degree of actual involvement of units in development are important, whereas units differed in their opinions. The quantitative study showed (nearly) consistent patterns of correlation coefficients in the expected direction for the majority of these characteristics. Notable exceptions included the level of conflict and mutual development from the franchisor perspective. However, moderate or strong correlations were frequently found with only some types of results, with different types of results for franchisors and units, and even with none of the results for one of the two groups. Moreover, although the qualitative study suggested that all these relationship characteristics were important, the quantitative study showed that there were more and stronger correlations for certain characteristics than for others. The quantitative study also revealed some unexpected correlations. These differences between the qualitative and quantitative findings suggest that perceptions about the role of particular relationship characteristics may not always reflect realities.

Overall, the integration of the qualitative and quantitative study demonstrates that unit actors (franchisees and managers) experience more satisfaction from working in the franchise when they experience well-attuned tasks, partnership, ample attention to their own needs and shared interests, little conflict, and ample space to voice their own ideas. They are also more satisfied when they feel well informed about internal and external developments, are more committed, and trust the franchisor. They experience satisfaction from working in such a relationship because they have space to autonomously fit the delivery of their care to local needs and feel respected regarding their professional ideas while simultaneously preventing reinvention of the wheel in developing improvements and valuable practices and realizing a synergy from franchise cooperation by using the strengths and inputs of others. For similar reasons, the survival chance and quality of care are rated higher when unit actors experience ample attention to their own needs and shared interests, strong commitment, and trust. As opposed to the qualitative study, all these characteristics were hardly correlated with other types of results from the unit perspective. This finding, among others, questions the qualitative perceptions that communication about external developments helps units anticipate changes and that conflict prevents cooperative activities. The absence of strong correlations between close cooperation and actual mutual involvement in development, on the one hand, and efficiency and quality on the other, also question the actual level of realized synergy and prevention of reinventing the wheel in such relationships from the unit perspective. Nevertheless, the satisfaction level of the units is an important indicator of success because studies have shown that satisfaction is key to the long-run viability of organizations and client satisfaction [29, 30].

Many of the preceding relationship characteristics also seem beneficial from the franchisor perspective for similar reasons, but for some other types of results. As in the unit group, strong commitment was related to quality and the promotion of work. In addition, a strong commitment to unit success was related to growth, survival and efficiency. This positive relationship with efficiency questions the correctness of the qualitatively perceived negative impact of franchisor commitment to units on the efficient division of tasks. The absence of a strong correlation between loyalty, competitive position and growth questions the importance of the qualitatively perceived acquisition advantage of loyalty. Higher levels of trust and attuned tasks benefit the franchisors’ perceived quality of care and survival. Survival also benefits from more participation opportunities for units, more actual joint development, and more attention to one another’s needs. Notably, actual development by units was negatively related to quality. The time left for units to provide care—a qualitatively perceived reason for the positive impact of more development by the franchisor—might explain this matter. Contrary to what was found for the units, it seems important for quality, efficiency, and survival from the franchisor perspective that units communicate about and share local developments because doing so prevents reinventing the wheel and helps develop a valuable business format. The unexpected positive correlation between conflict and quality questions the correctness of the qualitative perception that conflict distracts from cooperative, strengthening activities.

### Value of the Sequential Mixed Methods Design in Exploring Healthcare Franchises

Our study shows that we would have missed insights if we had not have used a sequential mixed methods design for our study in healthcare franchises. If we would have developed the cross-sectional survey based on theory from other industries rather than on the qualitative study, we would not have identified mutual involvement in development, sharing knowledge and experiences, commitment to the system and units, and trust in the respect for professional autonomy as important relationship characteristics in healthcare services. This conclusion demonstrates the relevance of using qualitative and quantitative methods sequentially—rather than simultaneously—to ensure methodological complementarity and cohesion. Without the qualitative study, we would also not have been able to understand the underlying reasons for the importance of various relationship characteristics. Without the quantitative study, we would have concluded that there were few differences between the groups of franchisors and units and that all relationship characteristics are perceived to be (equally) important. The quantitative study thus revealed nuances and quantifiable insights from a larger population.

### Strengths, Limitations and Directions for Future Research

This explorative study provided comprehensive insights on important relationship characteristics in healthcare franchises by combining two actor groups, multiple types of results, and qualitative and quantitative methods into a single study. The relevance of the research was reflected in the high survey response rate—nearly 70 percent of the units and more than 90 percent of the franchisors responded. However, our study has certain limitations that require further research.

First, our cross-sectional survey provided insight into the correlation between relationship characteristics and results but not into causality (direction). For example, the quantitative study showed moderate to strong correlations between commitment and trust elements, on the one hand, and results, on the other. The qualitative study suggested that these characteristics promote results. However, trust and commitment may also be high as a consequence of experiencing positive results. Research with a control group or longitudinal design is required to test for such causal relationships.

Moreover, our deliberate choice for data collection and analysis on the item level to ensure rich comprehensive insights and one-to-one integration between the qualitative and quantitative findings on relationship characteristics may have, to some degree, compromised the validity and reliability of the data because one item may not completely measure the concept and extreme responses on one item can more easily distort a conclusion. Additionally, the use of (mostly) non-validated items to ensure fit with our explorative qualitative findings may have compromised the validity of the study. The next step is to develop and use validated scales based on our findings in a larger population of franchisors and units.

Our sequential qualitative-quantitative design did not offer insight into the reasons for non-confirmed or unexpected correlations, such as the negative correlation, from the franchisor perspective, between development by units and promoting care. A follow-up qualitative study (sequential Qual-Quant-Qual design) might reveal explanations.

Finally, although our healthcare franchises in the Netherlands may share some characteristics with social healthcare franchises in low and middle-income countries—and this study can thus provide initial indications—confirmatory research in such settings is required.

### Conclusion

This explorative mixed methods study suggests that it is important for successful healthcare franchising to have communicatively open, committed, cooperative relationships in which franchisees and unit managers feel and trust that they have the opportunity to contribute ideas and articulate their needs to the franchisor. Such relationships allow the realization of synergy and local fit, alleviate professional resistance to implementation and prevent reinventing the wheel, which thereby helps ensure satisfaction in the units, survival from either the franchisor or unit perspective and, for most of these relationship characteristics, quality of care.

## Supporting Information

S1 Appendix Survey measures based on qualitative study. (DOC)

S1 Table Means and standard deviations relationship characteristics. (DOCX)

S2 Table Means and standard deviations perceived results. (DOCX)
