# Supplementary material for: Creating Advantages with Franchising in Healthcare: An Explorative Mixed Methods Study on the Role of the Relationship between the Franchisor and Units
Source: PLoS One. 2015 Feb 9;10(2):e0115829. doi: 10.1371/journal.pone.0115829 (PMC4321983; doi:10.1371/journal.pone.0115829)
Supplement: S1 Appendix — (DOC) [file pone.0115829.s001.doc]

**Appendix S1. Survey measures based on qualitative study**

The questionnaires are in Dutch. Below are the (non-validated) English translations.

**Relationship characteristics**

| **Concept** | **Survey items*, measured on 5-point Likert scale** | **Foundation of item** |
| --- | --- | --- |
| Close cooperation | - Relationship is characterized by partnership (rather than by a hierarchical type of relationship) - Relationship is characterized by attuned tasks and activities to achieve mutual goals - Relationship is characterized by respect and attention for one another’s needs and wishes | - [31] - Adapted from [18] - self-constructed |
| Sharing knowledge and experiences | - I frequently share knowledge, ideas, and experiences with other units in this system - I often share good ideas, improvements, innovations, and failures with the franchisor / central organization | - All self-constructed |
| Mutual involvement in development of improvements and innovations | - Opportunity to participate:   - There is sufficient opportunity to participate in the development of new strategies, policies, products, services, and work methods in this franchise system - Actual mutual involvement in development:   - I frequently help thinking about improvements and innovations in this franchise system   - I develop many improvements and innovations in my own unit(s)   - The franchisor / central organization develops many ideas for improving and renewing policies, products, services, and work methods | - Adapted from [17] - Items 2, 3, 5 self-constructed |
| Commitment | - I defend this franchise system when outsiders criticize it - I am willing to spend time and resources to make the entire franchise system successful (e.g., by going to joint meetings, by helping others) - I am willing to spend time and resources to make my unit(s) successful | - All adapted from [3, 17] |
| Trust | - I trust the franchisor / central organization / we trust the units (FO-unit trust) - I trust that the franchisor / central organization respects my professional autonomy and expertise | - Item 1 adapted from [31] - Self-constructed |
| Mutual communication | - I am sufficiently informed about what the franchisor / central organization has done with my ideas or suggestions - The franchisor / central organization keeps me sufficiently informed about internal developments - The franchisor / central organization keeps me sufficiently informed about external developments - I keep the franchisor / central organization sufficiently informed about the opportunities and threats I encounter with my unit | - All adapted from [3, 18] |
| Conflict & focus on own interests | - The relationship with the franchisor has gone through unstable and conflicting stages - People in this franchise system are primarily focused on their own interests | - [18] - Adapted from [32] |

* These are the unit survey items. The franchisor items were similar but formulated from the franchisor perspective (e.g., ‘I frequently help thinking about improvements and innovations in this franchise system’ was formulated as ‘The units frequently help thinking about improvements and innovations in this franchise system’).

**Perceived results**

| **Concept** | **Survey items, measured on 5-point Likert scale** | **Foundation of item** | **Cronbach’s Alpha or inter-item** |
| --- | --- | --- | --- |
| Competitive position | - We have a strong position with reference to other care providers that provide the same type of care - We have a strong position with respect to the financiers of our care | - Adapted from [14] | - Franchisor : Alpha .66, Inter-item .50 - Unit : Alpha .65, Inter-item .48 |
| Financial performance | - The financial performance (turnover, positive result, production) is good | - Adapted from [25, 33] | n.a. |
| Efficiency | - We provide care efficiently - We innovate and improve efficiently | - Self-constructed | - Franchisor: Alpha. 59, Inter-item .43 - Unit: Alpha .77, inter-item .63 |
| Survival | - Through participating in this franchise my likelihood of survival is greater in the current care environment^B^/ Franchisees are more likely to survive in the current care environment by participating in this franchise^A^ - Through this franchise, our likelihood of survival as a franchisor organization is greater in the current care environment^A^ | - Self-constructed | - Franchisor: alpha .84, inter-item .78 - Unit: n.a. |
| Growth | - The number of units in this system has grown^A^ - We can grow further in the coming years^A^ | - Self-constructed | - Franchisor: alpha .73, inter-item .58 - Unit: n.a. |
| Quality of care | - Good quality of care (scale)   - The patients / clients are satisfied with the care we provide   - I can provide the care that I want to provide from a professional perspective^B^ / care providers can provide the care in the manner they want to provide it from a professional perspective^A^   - The care we provide is high quality - Promote care (ultimate question), (rated on a scale 0 – 10 (0 = very unlikely, 10 = very likely):   - To what extent would you recommend colleagues, friends, and family to become a customer / patient / client of this franchise if they need this type of care? | - First three items self-constructed - Net Promotor Score; adapted from [18, 34] | - Franchisor: alpha .72 - Unit: alpha .79 |
| Satisfaction | - Satisfied with work in franchise (scale)   - I am satisfied with this franchise^B^   - I intend to remain in this system in the coming years ^B^   - I feel more relieved through this franchise compared to what I would have felt in another job or as an independent entrepreneur^B^   - I am satisfied with the possibilities I have to bring in my own ideas and visions^B^   - I feel satisfied through working in this franchise^B^ - Promote work in franchise (ultimate question), (rated on a scale 0 – 10 (0 = very unlikely, 10 = very likely)   - To what extent would you recommend colleagues, friends, and family to become a franchisee within this franchise? | - - First two ítems: [3, 18, 34]   - Other items: adapted from [4, 35, 36]   - Net Promotor Score; adapted from [18, 34] | - - Unit: alpha .84 (NB: without intention to remain due to missing values) |

**^A^ =** in franchisor questionnaire only, **^B^ =** in unit (franchisee and manager) questionnaire only

**References (see the main article for references [1] to [30] )**

1. Herz MF, Hutzinger C, Seferagic H, Windsperger J (2013) Trust, decentralization and network performance: The case of franchising. Proceedings of the 27^th^ annual ISOF conference, 13-16 March 2013, Zhuhai, China.
2. Gassenheimer JB, Baucus DB, Baucus MS (1996) Cooperative arrangements among entrepreneurs: an analysis of opportunism and communication in franchise structures. J Bus Res 36: 67-79.
3. Minguela-Rata B, López-Sánchez JI, Rodríguez-Benavides MC (2009) The effect of knowledge complexity on the performance of franchise systems in the service industries: an empirical study. Service Business 3: 101-115.
4. Chiou J, Hsieh C, Yang C (2004). The effect of franchisors’ communication, service assistance, and competitive advantage on franchisees’ intentions to remain in the franchise system. J Small Bus Manage 42: 19-36.
5. Morrison KA (1996) An empirical test of a model of franchisee job satisfaction. J Small Bus Manage 34: 27-41.
6. Wijk, K.P. van (2007). The service care chain: the influence of service and HRM on the realization of demand-driven care by care organisations (doctoral dissertation, in Dutch). Erasmus University Rotterdam.
