# Supplementary material for: Creating Advantages with Franchising in Healthcare: An Explorative Mixed Methods Study on the Role of the Relationship between the Franchisor and Units
Source: PLoS One. 2015 Feb 9;10(2):e0115829. doi: 10.1371/journal.pone.0115829 (PMC4321983; doi:10.1371/journal.pone.0115829)
Supplement: S1 Table — (DOCX) [file pone.0115829.s002.docx]

**Table S1: Means and standard deviations relationship characteristics**

(5-point Likert scale, 1=completely disagree, 5=completely agree)

|  | | **Franchisor**  **(n = 40)** | |  | **Unit actors**  **(n = 346)** | | | |
| --- | --- | --- | --- | --- | --- | --- | --- | --- |
|  |  | Mean | SD | Min.-Max | Mean | SD | Min-Max. |  |
| **Close cooperation** | Attention needs | 4.04 | .68 | 2-5 | 3.84 | .91 | 1-5 |  |
|  | Partnership | 4.11** | .68 | 2-5 | 3.60** | 1.01 | 1-5 |  |
|  | Attuned tasks | 3.70 | .82 | 2-5 | 3.54 | .91 | 1-5 |  |
| **Knowledge sharing** | Between units | 3.68 | .97 | 2-5 | 3.54 | .95 | 1-5 |  |
|  | With franchisor | 3.43 | .96 | 2-5 | 3.59 | .86 | 1-5 |  |
| **Mutual involvement in developing improvements and innovations** | Franchisor development | 4.13*** | .72 | 2-5 | 3.36*** | .9 | 1-5 |  |
|  | Opportunity participation units | 4.18*** | .72 | 2-5 | 3.53*** | .97 | 1-5 |  |
|  | Units frequently think along | 3.78** | .83 | 2-5 | 3.28** | .96 | 1-5 |  |
|  | Development by units | 3.13*** | .89 | 2-5 | 3.77*** | .71 | 1-5 |  |
| **Mutual commitment** | Loyalty – defend criticize | 4.63*** | .54 | 3-5 | 4.00*** | .77 | 1-5 |  |
|  | Willingness to make system successful | 4.60*** | .59 | 3-5 | 4.08*** | .81 | 1-5 |  |
|  | Willingness to make unit successful | 4.58 | . 59 | 3-5 | 4.49 | .54 | 2-5 |  |
| **Trust** | Franchisor – unit trust | 4.15 | .58 | 3-5 | 3.88 | .90 | 1-5 |  |
|  | Trust respect autonomy | 4.25 | .54 | 3-5 | 4.03 | .77 | 1-5 |  |
| **Mutual communication** | By unit | 3.50* | .82 | 2-5 | 3.80* | .76 | 1-5 |  |
|  | External developments | 3.95** | .64 | 2-5 | 3.47** | .96 | 1-5 |  |
|  | Internal developments | 3.90** | .59 | 2-5 | 3.46** | .95 | 1-5 |  |
|  | Done with ideas | 3.80*** | .76 | 2-5 | 3.26*** | .89 | 1-5 |  |
| **Conflict, opportunistic behavior** | Conflict | 3.38*** | 1.09 | 1-5 | 2.66*** | 1.25 | 1-5 |  |
|  | Focus own interests | 2.51# | 1.00 | 1-5 | 2.81# | 1.04 | 1-5 |  |

Significant differences between groups: # P < .10; * P <.05; ** P<.01; *** P=.000 (Mann Whitney-U tests)
