# Supplementary material for: Creating Advantages with Franchising in Healthcare: An Explorative Mixed Methods Study on the Role of the Relationship between the Franchisor and Units
Source: PLoS One. 2015 Feb 9;10(2):e0115829. doi: 10.1371/journal.pone.0115829 (PMC4321983; doi:10.1371/journal.pone.0115829)
Supplement: S2 Table — (DOCX) [file pone.0115829.s003.docx]

**Table S2: Means and standard deviations perceived results**

(5-point Likert scale, 1=completely disagree, 5=completely agree)

|  | | **Franchisor**  **(n = 40)** | | | **Unit actors**  **(n = 346)** | | |
| --- | --- | --- | --- | --- | --- | --- | --- |
|  |  | Mean | SD | Min-max | Mean | SD | Min-max |
| **Financial results good** | | 3.44 | 1.05 | 1-5 | 3.64 | 1.01 | 1-5 |
| **Efficiency of care and innovation** | | 4.24 | .58 | 3-5 | 4.05 | .69 | 2-5 |
| **Quality of care** | Good quality of care (scale) | 4.49** | .42 | 4-5 | 4.25** | .52 | 2-5 |
|  | Promote care (11-point scale) | 9.10*** | .80 | 7-10 | 8.16*** | 1.68 | 0-10 |
| **Competitive position** | | 4.21** | .64 | 2-5 | 3.88** | .81 | 1-5 |
| **Survival chance of participants** | | 4.36** | .71 | 2-5 | 3.98** | .86 | 1-5 |
| **Satisfaction** | Satisfied with work in the franchise (scale) | - | - | - | 3.79 | .77 | 1-5 |
|  | Promote work in franchise (11-point scale) | 8.29*** | 1.70 | 1-10 | 6.96*** | 2.04 | 0-10 |
| **Growth system** | | 4.36 | .74 | 3-5 | - | - | - |

Significant differences between groups: # P < .10; * P <.05; ** P<.01; *** P=.000 (Mann Whitney-U tests)
